# Global Deficiency of Alzheimer’s Disease Risk Gene *Il1rap* Reduces Pathological Tau in a Mouse Model of Systemic Inflammation

**DOI:** 10.1080/17590914.2025.2598310

**Published:** 2025-12-06

**Authors:** Somayeh Dadras, Kiran Bhaskar

**Affiliations:** a Department of Molecular Genetics and Microbiology, University of New Mexico, Albuquerque, NM, USA; b Department of Neurology, University of New Mexico, Albuquerque, NM, USA

**Keywords:** Alzheimer’s disease, interleukin-1, interleukin-1 receptor, interleukin-1 receptor accessory protein, IL-1RAcP, tauopathies

## Abstract

Brain inflammation is strongly associated with neurodegeneration in Alzheimer’s disease (AD) and related tauopathies. We have previously demonstrated that microglia-derived interleukin-1β (IL-1β) induces tau hyperphosphorylation in a cell-autonomous manner and depends on activating the IL-1 receptor (IL-1R1) signaling pathway. IL-1 receptor accessory protein (IL-1RAcP) is a co-receptor for IL-1R1 and is essential for the IL-1R1 receptor function and downstream signaling. Genome-wide association studies have identified several single-nucleotide polymorphisms (SNPs) in the *IL1RAP* gene that have been shown to increase AD risk. Here, we demonstrate that global and neuron-specific isoform deficiency of IL-1RAcP regulates hyperphosphorylated tau levels in a lipopolysaccharide (LPS)-induced mouse model of systemic inflammation. Notably, while global *Il1rap^-/-^* reduced pS202(AT8) and pT231 (AT180) tau levels, neuron-specific IL-1RAcP (IL-1RAcPb) deficiency specifically increased total tau levels. Together, these results suggest that IL-1RAcP is an important regulator of tau hyperphosphorylation relevant to AD and related tauopathies.

## Introduction

Tauopathies are a group of neurodegenerative diseases characterized by abnormal tau protein accumulation in the brain, leading to cognitive and motor dysfunction. A hallmark of these disorders, including Alzheimer’s disease (AD), progressive supranuclear palsy, and corticobasal syndrome, is the presence of pathological tau inclusions associated with neuronal death and disease progression (Creekmore et al., 2024; Yoshiyama et al., 2007). Neuroinflammation plays a pivotal role in tau protein aggregation and deposition. Glial cells, including microglia and astrocytes, release pro-inflammatory cytokines that drive tau hyperphosphorylation, a critical step in tau aggregation (Davidson et al., 2023). Chronic neuroinflammation perpetuates a cycle of tau aggregation and neuronal damage, contributing to tauopathies such as AD (Langworth-Green et al., 2023; Metcalfe & Figueiredo-Pereira, 2010). While microglia can clear tau aggregates, their sustained activation often enhances tau pathology, exacerbating neurodegeneration (Dani et al., 2018; Davidson et al., 2023). Interleukin-1 beta (IL-1β), a key cytokine in neuroinflammation, exacerbates tau pathology by activating pathways such as p38 Mitogen Activated Protein kinase (p38 MAPK), glycogen synthase kinase-3β (GSK3β), and cyclin-dependent kinase 5(CDK5) (Kitazawa et al., 2005), which promote tau phosphorylation (Mendiola & Cardona, 2018). Chronic IL-1β overexpression in murine models leads to 2–4-fold increases in phospho-tau levels, linking IL-1β directly to tau hyperphosphorylation and microglial activation (Ghosh et al., 2013). Furthermore, IL-1β overexpression can lead to neurodegeneration (Rossi et al., 2014) and cognitive impairment (Rachal Pugh et al., 2001), while blocking IL-1β signaling attenuates tau pathology and rescues memory deficits in animal models (Kitazawa et al., 2011).

**Figure 1.**
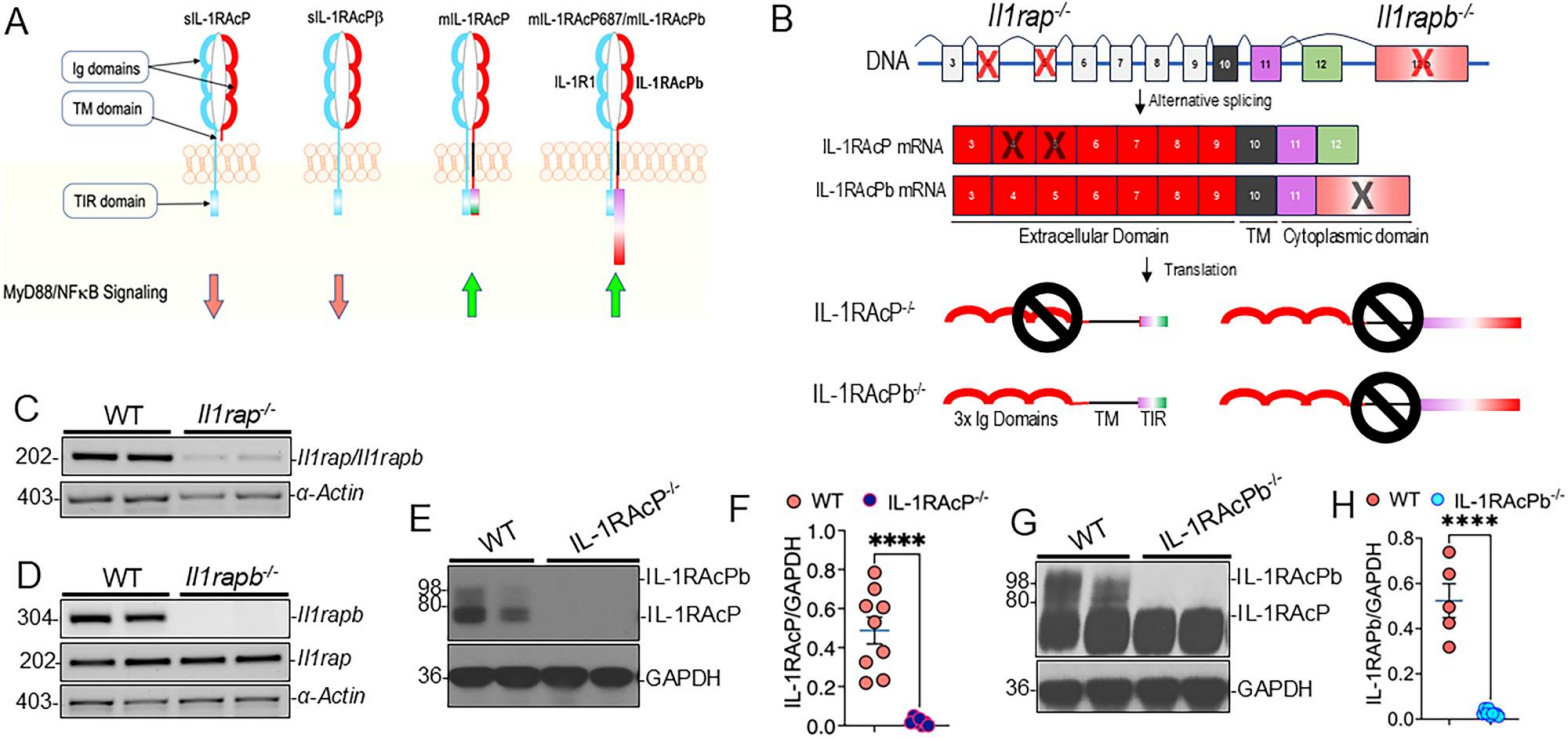
IL-1RAcP and validation of IL-1RAcP knockout. (A) Schematic showing different isoforms of IL-1RAcP with immunoglobulin (Ig) and transmembrane (TM) and Toll-Interleukin-Receptor (TIR) domains. Also shown are which isoforms are productive in transducing IL-1R1 intracellular signaling through MyD88/NF-κB pathway. s: soluble; m: membrane bound. Green arrow is indicative of activation of MyD88/NF-κB signaling. Red arrow indicates b signaling. (B) Schematic showing exons 4 and 5 in *Il1rap^-/-^* animals and exon 12b in *Il1rapb^-/-^* mice are deleted. Details are in [Ref # 40 and 17] respectively. Ig = Immunoglobulin; TM = Transmembrane; TIR = Toll-IL1R-domain. (C and D) PCR showing amplification of *Il1rap* and *Il1rapb* gene in *Il1rap^-/-^* and *Il1rapb^-/-^* mice respectively. α-actin is used as housekeeping control. (E) Western blot showing loss of IL-1RAcP and IL-1RAcPb bands at ∼80 KDa and ∼98 KDa in the hippocampal lysates of *Il1rap^-/-^* mice compared to non-transgenic wild type (WT) mice. (F) Quantification of the ratio of IL-1RAcP/GAPDH showing significant reduction. (G) Western blot showing the deletion of IL-1RAcPb in the hippocampal lysates of *Il1rapb^-/-^* mice compared to WT mice. (H) Quantification of the ratio of IL-1RAcP/GAPDH and IL-1RAcPb/GAPDH showing significant reduction in IL-1RAcP levels respectively. Data presented as mean ± SEM; n = 5–9 mice/group; ****p < 0.0001 unpaired *t* test).

Key mediators of IL-1β signaling are the IL-1 receptor 1 (IL-1R1) and its co-receptor, IL-1 receptor accessory protein (IL-1RAcP). IL-1R1 requires IL-1RAcP to function, and their interaction activates Nuclear Factor-κB (NF-κB) or p38 MAPK, leading to cytokine release (Sims & Smith, 2010). The human *IL1RAP* gene is located on chromosome 3q28 and contains 12 exons (Jensen et al., 2000). Alternative splicing of IL1RAP mRNAs results in four different splice variants (Figure 1A and B): (1) 570 amino acid membrane form – membrane-bound (m)IL-1RAcP; (2) 687 amino acid membrane form – mIL-1RAcP687 (Sims & Smith, 2010); (3) 356 amino acid soluble (s) form – sIL-1RAcP; (4) 346 amino acid soluble form of sIL-1RAcPβ (Alkam et al., 2008; Lu et al., 2008). These isoforms differ with respect to their extracellular immunoglobulin (Ig), transmembrane (TM), and intracellular Toll/interleukin-1 receptor (TIR) homology domains. Both membrane forms of IL-1RAcP have been shown to positively activate IL-1/IL-1R1 signaling cascade; however, the soluble forms lacking intracellular domains negatively affect IL-1/IL-1R1 signaling (Jensen et al., 2000). Interestingly, recent studies have suggested that mIL-1RAcP687 (also called as IL-1RAcPb or ‘AcPb’) is exclusively expressed by CNS neurons (Smith et al., 2009). Together, these results suggest a complexity of IL-1/IL-1R1/IL-1RAcP signaling, which has never been studied with respect to microglial neuroinflammation during the pathogenesis of non-AD tauopathies.

We have previously demonstrated that lipopolysaccharide (LPS), which causes neuroinflammation through toll-like receptor-4 (TLR-4), can trigger tau phosphorylation in neurons (Batista et al., 2019; Bhaskar et al., 2010). Inducing inflammation in a manner cell-autonomous to microglia via *Cx3cr1* deficiency and LPS induced tau hyperphosphorylation, which was dependent on IL-1β/IL-1R signaling pathway (Bhaskar et al., 2010). Adoptive transfer of *Cx3cr1-*deficient microglia from hTau donor mice into the brains of non-transgenic mice resulted in exacerbation of tau pathology in an IL-1R1-dependent manner (Maphis et al., 2015). We have also shown that pathologically modified tau, in turn, serves as a damage-associated molecular pattern (DAMP) to trigger IL-1β activation in microglia (Jiang et al., 2021). Neutralizing pathological tau with a vaccine or genetic deficiency of inflammasomes reduced IL-1β activation, neuroinflammation, tau hyperphosphorylation, and improved memory in different mouse models of tauopathy (Jiang et al., 2021; Maphis et al., 2025). Since IL-1R1 requires IL-1RAcP to mediate the effects of IL-1β, it is unclear if IL-1RAcP plays any role identical to IL-1R1 in driving tau pathology. This is important to understand because IL-1RAcP also serves as a co-receptor for other pro-inflammatory cytokines (e.g., IL-1α, IL-33 (Sims & Smith, 2010; Fields et al., 2019) and IL-36 (Fields et al., 2019; Yuan et al., 2019; Masoumi et al., 2020), which are implicated in neurodegeneration and Alzheimer’s disease-related dementias (ADRD) (Allan et al., 2016; Brough & Denes, 2015; Byrne et al., 2021; Chapuis et al., 2009; Chen et al., 2021; Dursun et al., 2015; Italiani et al., 2018; Luheshi et al., 2011; Reverchon et al., 2020; Xiong et al., 2014; Zhang et al., 2019). Furthermore, prior genome-wide association studies (GWAS) identified *IL1RAP* as an AD risk gene (Jones et al., 2010). Specifically, the *IL1RAP* single-nucleotide polymorphism (SNP) rs12053868 is linked to an increased risk of progression from mild cognitive impairment to AD, accompanied by temporal cortex atrophy (Ramanan et al., 2015). Another SNP (rs9877502) has been significantly correlated with elevated phosphorylated tau (pTau) levels in cerebrospinal fluid (CSF) of AD patients (Cruchaga et al., 2013). These findings suggest IL-1RAcP may contribute to tau pathology in tauopathies. However, its precise role in inflammation-driven tauopathies remains unclear. This study aims to elucidate whether IL-1RAcP elimination can mitigate neuroinflammation and tau pathology. Here, we used a global and neuron-specific isoform of IL-1RAcP knockout mice to determine if IL-1RAcP deficiency has any role in the LPS-induced tau pathology. Global IL-1RAcP knockout mice show reduced LPS-induced tau pathology in the hippocampus. Interestingly, genetic deletion of only neuron-specific IL-1RAcPb isoform also shows elevated total tau levels and a modest reduction of hyperphosphorylated tau in the hippocampus.

## Materials and Methods

Details of materials used, catalog numbers, and sources are included in the Supplemental Table S1.

### Animals

*Il1rap^-/-^* and *Il1rapb^-/-^* mice were obtained from Dr. James Krueger (Washington State University). In *Il1rap^-/-^* mice, exons 4 and 5 are deleted (Figure 1B), which results in the loss of the first and part of the second immunoglobulin-like (Ig-like) domains in all IL-1RAcP protein isoforms (Cullinan et al., 1998; Smith et al., 2009). In contrast, *Il1rapb^-/-^* mice have a deletion in the last exon (12b) of *Il1rap* (Figure 1B), which encodes the intracellular domain of Il-1RAcPb, therefore resulting in the knockout of only the IL-1RAcP isoform in these animals (Smith et al., 2009) (Figure 1B). Both lines are in the C57Bl/6J background. All animal procedures were conducted in compliance with the U.S. National Institutes of Health guidelines for the care and use of laboratory animals and were approved by the University of New Mexico Institutional Animal Care and Use Committee (protocols 22-201247-B-HSC and 24-201557-HSC). Mice were housed under specific pathogen-free (SPF) conditions on a 12-hour light/dark cycle, with unrestricted access to food and water. Animals were kept in 85 in^2^ ventilated microisolator cages containing autoclaved TEK Fresh crinkle bedding and were provided with environmental enrichment, including tissue paper and a raised penthouse insert.

### Lipopolysaccharide Administration

A single intraperitoneal (i.p.) injection of LPS from Escherichia coli O55:B5 was administered at a dose of 3.5 mg/kg body weight to 20-22-month-old non-transgenic wild-type (WT), *Il1rap^-/-^* and *Il1rapb^-/-^* mice. This age is equivalent to a human age of 60–64 years (When are mice considered old?, 2023) . At this age, mice typically show an appreciable amount of age-related basal tau phosphorylation, and we wanted to understand how tau pathology at this age is impacted by IL-1RAP deficiency. Twenty-four hours post-injection, mice were euthanized, and brain tissue was harvested as described below. Previous studies from our lab have demonstrated that LPS doses of 3 mg/kg body weight or higher trigger tau phosphorylation in the hippocampus of wild-type mice (Weston et al., 2021; Bemiller et al., 2018).

### Tissue Collection and Processing

Mice were anesthetized and perfused transcardially with cold 0.125 M phosphate buffer (PB; pH 7.4), after which brains were extracted. The right hemispheres were dissected into the cortex, hippocampus, and rest of the brain. Each region was weighed, and samples were immediately frozen in liquid nitrogen for subsequent analysis. The left hemispheres were placed in 4% paraformaldehyde (PFA) prepared in PB for fixation. Following a 24-hour fixation period, the PFA solution was removed, and tissues were transferred to a cryoprotectant solution for another 24 hours. Brains were then sectioned sagittally (30 μm thick) using a cryomicrotome, put in cryostorage solution, and stored at –20 °C until further processing for immunohistochemistry.

### RNA Extraction and Gene Expression Analysis

RNA was extracted from the rest of the right hemisphere (after dissecting out the cortex and hippocampus) using TRIzol reagent, and cDNA was synthesized using the High-Capacity cDNA Reverse Transcription Kit. Standard PCR was performed to confirm the knockout in Il1rap-/- and *Il1rapb^-/-^* mice using specific primer sequences listed in Supplemental Table S1 and OneTaq Master Mix. α-Actin was used as the housekeeping control. PCR conditions were as follows: initial denaturation at 95 °C for 5 min, followed by 35 cycles of 95 °C for 30 s, annealing at 61 °C for Il1rap and *Il1rapb* primers or 58 °C for α-actin for 30 s, and extension at 72 °C for 30 s, with a final extension at 72 °C for 7 min. PCR products were separated on a 1.5% agarose gel, stained with GelRed, and visualized using a ChemiDoc imaging system.

### Western Blotting

Hippocampal samples were homogenized using Tissue Protein Extraction Reagent (TPER) supplemented with protease and phosphatase inhibitors. The lysates were then sonicated and centrifuged at 13,700 × g for 30 minutes at 4 °C. The resulting supernatant was combined with lithium dodecyl sulfate (LDS) sample buffer and reducing agent, then heated at 95 °C for 15 minutes. Proteins were separated on 4–12% Bis-Tris NuPAGE gels with 2-(N-morpholino)ethanesulfonic acid or MES buffer (for tau) and NuPAGE 8% Bis-Tris gel with (3-(N-morpholino)propanesulfonic acid) or MOPS buffer (for IL-1RAcP knockout validation) and transferred to PVDF membranes overnight. Following transfer, membranes were washed, blocked with either 3% BSA or 5% non-fat milk in PBS, and incubated overnight with primary antibodies with the following dilutions: IL-1RAP (1:2000), phosphorylated tau antibodies: AT180 (1:5000), PHF-1 (1:10,000); AT8 (1:10,000); Tau5 (1:10000); GAPDH (1:100,000). After washing, the membranes were exposed to corresponding HRP-conjugated secondary antibodies for one hour, developed using enhanced chemiluminescence (ECL) reagent, and visualized on X-ray film. Films were scanned and immunoreactive bands were analyzed using AlphaEaseFC^™^ software.

### Immunohistochemistry

Free-floating brain sections (30 µm) were subjected to immunohistochemistry following standard protocols. Antigen retrieval was performed using a sodium citrate buffer, followed by quenching of endogenous peroxidase activity with 3% hydrogen peroxide in PBST. Non-specific binding was blocked using 5% normal goat serum in PBS containing Triton X-100. Sections were incubated overnight at 4 °C with the AT8 primary antibody (1:250), then exposed to a biotinylated secondary IgG antibody (1:200) for 1 hour. Afterward, sections were treated with an avidin-biotin enzyme complex for 2 hours at room temperature. Detection of immunolabeling was achieved using SIGMAFAST 3,3′-Diaminobenzidine (DAB) substrate without metal enhancement. Labeled sections were mounted onto glass slides, dehydrated through a graded ethanol series, cleared in xylene, and cover slipped with Permount mounting medium. Bright-field images were captured with an Olympus BX-51 microscope (Olympus America Inc.), equipped with an Optronics digital camera and Picture Frame image capture software (Optronics).

### Statistics

Statistical differences between groups were assessed using unpaired t-test. Analyses were conducted using GraphPad Prism^®^ software (version 10.4.1). Results are expressed as mean ± standard error of the mean (SEM), with sample sizes detailed in the corresponding figure legends.

## Results

### Validation of IL-1RAcP Knockout in *Il1rap^-/-^* and *Il1rapb^-/-^* Mice

IL-1RAcP–deficient mice were validated for the knockout using RT-PCR and Western blot analyses (Figure 1C, E–F). Following RNA extraction and cDNA synthesis, PCR was performed to assess *Il1rap* gene expression in wild-type and knockout mice. The *Il1rap*^-/-^ mice lack exons 4 and 5, which are common to all resulting isoforms; therefore, these mice do not express either IL-1RAcP or IL-1RAcPb (Figure 1B–C). The expected 202 base pairs (bp) PCR product corresponds to amplification across exons 4 and 5; hence, its absence confirms knockout of both isoforms (Figure 1C).

In contrast, *Il1rapb*^-/-^ mice carry a deletion in exon 12b, which is specific to the IL-1RAcPb isoform, resulting in selective loss of this neuronal variant while retaining expression of the global isoform (IL-1RAcP) (Figure 1B and C). The 304 bp PCR product shown in Figure 1D represents amplification across exon 12b.

Western blot analysis of brain homogenates from wild-type mice revealed two bands at approximately 80 and 98 kDa corresponding to IL-1RAcP and IL-1RAcPb, respectively, which were absent in the *Il1rap*^-/-^ mice (Figure 1E). Quantification confirmed a complete loss of IL-1RAcP protein (Figure 1F). Similarly, Western blot analysis of *Il1rapb*^-/-^ mice and their wild-type controls showed two distinct bands in wild-type brains that were absent in the *Il1rapb*^-/-^ mice (Figure 1G). Quantification of the 98 KD band in both groups confirmed the knockout of IL-1RAcPb in the *Il1rapb*^-/-^ mice (Figure 1H). While we believe that Western blot analyses confirmed *Il1rap*^-/-^ and *Il1rapb*^-/-^ mice, because the 80 kDa and 90 kDa bands are difficult to resolve well even with 8% Bis-Tris gel with longer running time, we recommend validating the knockout based on the RT-PCR in addition to Western blot.

### Global *Il1rap^-/-^* Mice Show Reduced Hyperphosphorylated Tau Levels Compared to Wild-Type Mice

**Figure 2.**
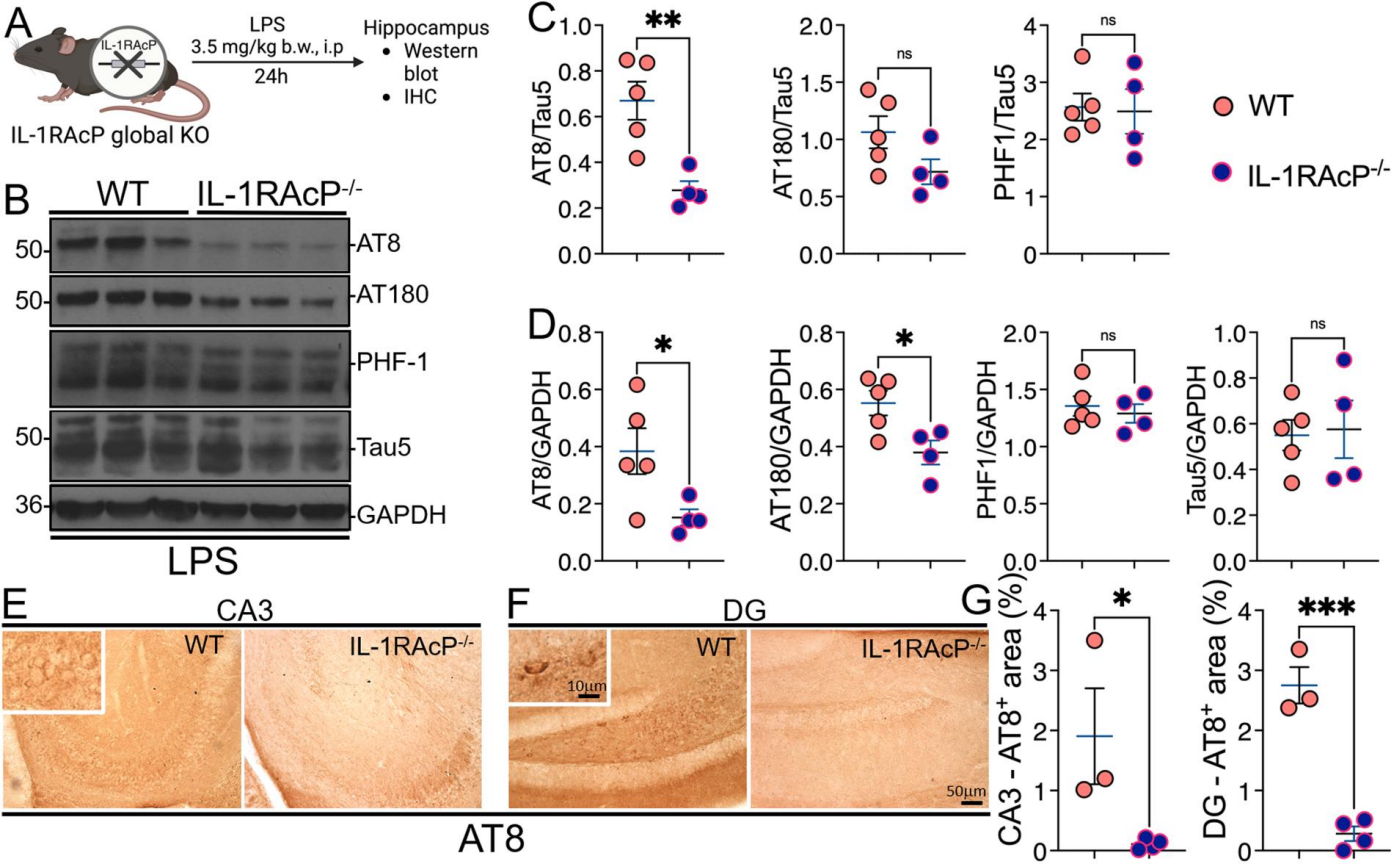
Global *Il1rap^-/-^* mice shows reduced hyperphosphorylated tau levels compared to wild type mice. (A) Schematic showing global knock out of IL-1RAcP. (B) Western blot showing levels of AT8^+^, AT180^+^, and PHF-1^+^ hyperphosphorylated tau and total tau in LPS (3.5 mg/kg b.w; i.p; 24 h) injected wild type and IL1RAcP ^-/-^ mice. (C and D) Quantification of the ratio of AT8, AT180, PHF-1 to Tau5 (C) and GAPDH (D) showing significant reduction in AT8/Tau5 and AT180/GAPDH. (E and F) Immunohistochemistry images of the CA3 and dendrite gyrus (DG) showing reduced levels of AT8 hyperphosphorylated tau in IL1RAcP ^-/-^ mice compared to wild type mice. (G) Quantification of the percentage of AT8^+^ areas in CA3 and DG. Data presented as mean ± SEM; n = 3–5 mice/group; *p < 0.01, ***p < 0.001, ***p < 0.0001 unpaired *t* test). Scale bars 10 µm (inset); 50 µm (outside).

Wild-type and *Il1rap^-/-^* mice were injected with LPS and used as an inflammation-induced tauopathy model to assess changes in hyperphosphorylated tau levels in the brain (Figure 2A). LPS-injected IL-1RAcP deficient mice significantly reduced pS202/pS205 (AT8)/Tau5, AT8/GAPDH and pT231 (AT180)/GAPDH ratios compared to LPS-injected wild-type mice (Figure 2B, C and D). To further validate these results, we performed immunohistochemistry analysis for AT8 in the CA3 and dentate gyrus in the LPS-injected IL1-RAcP deficient and WT mice. We observed AT8^+^ neurons in both CA3 and DG of LPS-injected WT mice, which was significantly reduced in LPS-injected IL-1RAcP deficient mice (Figure 2E and F). Notably, quantitative morphometric analyses showed a statistically significant reduction in the AT8^+^ area in both CA3 and DG of LPS-injected *Il1rap^-/-^* mice compared to LPS-injected WT controls (Figure 2G). These results suggest that global knockout of IL-1RAcP reduced phosphorylated tau levels (specifically AT8) in a mouse model of systemic inflammation and tau pathology.

### Neuron-Specific IL-1RAcP Deficient Mice Show Increased Elevated Tau5 Levels Compared to Wild-Type Mice

**Figure 3.**
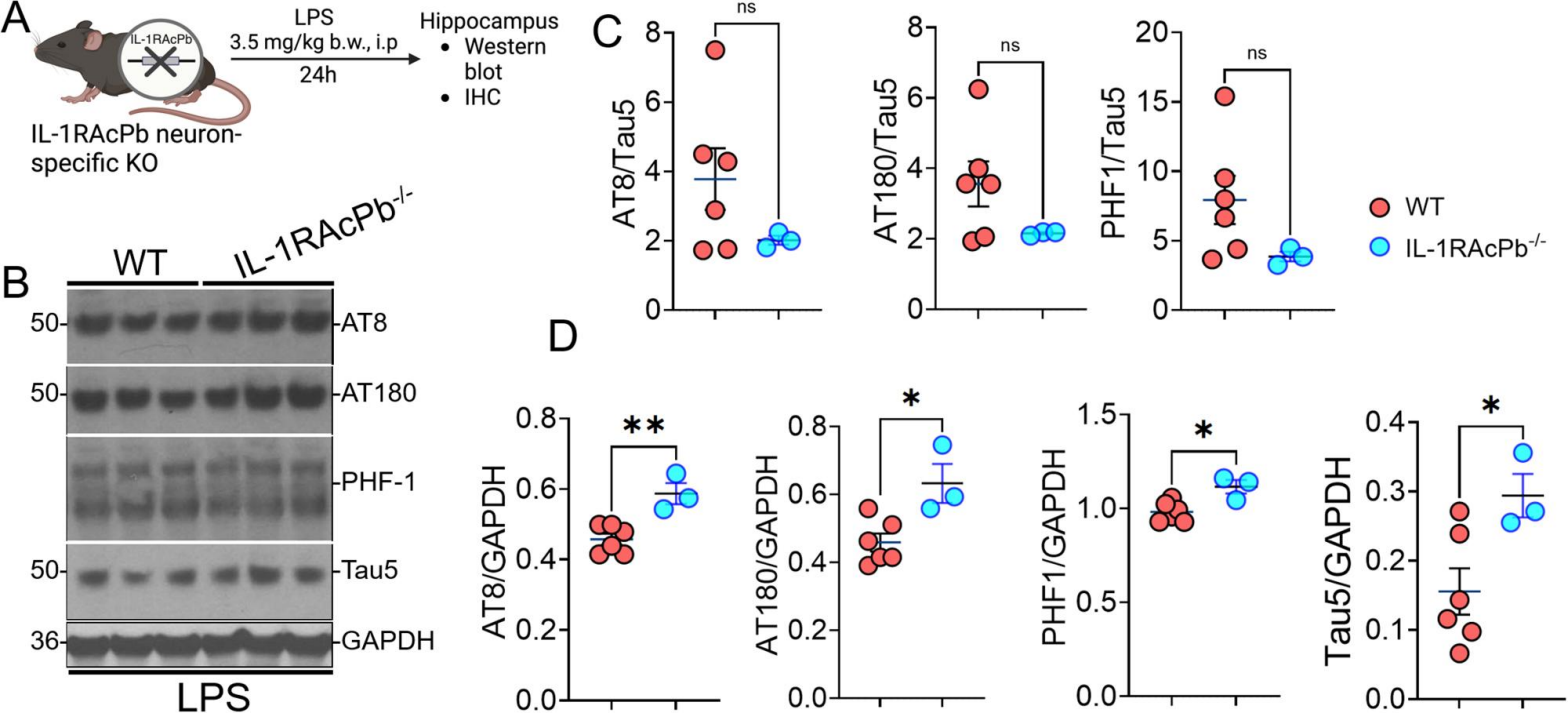
Neuron-specific IL-1RAcP deficient mice show reduced hyperphosphorylated tau levels compared to wild type mice. (A) Western blot showing levels of AT8^+^, AT180^+^, and PHF-1^+^ hyperphosphorylated tau and total tau in LPS (3.5 mg/kg b.w; i.p; 24 h) injected wild type and IL1RAcPb^-/-^ mice. (B and D) Quantification of the ratio of AT8, AT180, PHF-1 to Tau5 (C) and GAPDH (D) showing significant reduction in PHF-1/Tau5 and AT8/GAPDH, AT180/GAPDH, and Tau5/GAPDH. Data presented as mean ± SEM; n = 3–6 mice/group; *p < 0.01, **p < 0.001, unpaired *t* test).

Next, to determine whether deletion of neuron-specific IL-1RAcPb isoform has any effect on LPS-induced tau phosphorylation, we injected IL-1RAcPb knockout (*Il1rapb^-/-^*) mice with LPS and analyzed tau phosphorylation levels after 24 hours (Figure 3A). Unlike global IL-1RAcP knockout mice, IL-1RAcPb knockout mice showed a modest reduction in AT8/Tau5, AT180/Tau5 and PHF-1/Tau5 ratios upon LPS challenge compared to LPS-injected WT mice (Figure 3B and C). Notably, when the AT8, AT180, and PHF-1 ratio were plotted against GAPDH, we observed a significant increase in the AT8/GAPDH, AT180/GAPDH, and PHF-1/GAPDH levels in LPS-injected IL-1RAcPb knockout mice compared to LPS-injected WT mice. This was likely due to a significant increase in Tau5/GAPDH ratio in LPS-injected IL-1RAcPb knockout mice (Figure 3D). We performed immunohistochemistry analyses, but there were no major differences observed in the levels of hyperphosphorylated tau between LPS injected WT and LPS injected IL-1RAcPb  groups (not shown). Therefore, we conclude that the neuron-specific IL-RAcPb deletion increases the Tau5 levels upon LPS challenge.

## Discussion

In this study, we used global and neuron-specific IL-1RAcP knockout mice to determine if IL-1RAcP deficiency plays a role in LPS-induced tau pathology. Our findings indicate that LPS-induced tau pathology in the hippocampus is reduced in global IL-1RAcP knockout mice. Notably, deleting only the neuron-specific IL-1RAcPb isoform, increased total tau levels but did not alter AT8/Tau5, AT180/Tau5, or PHF-1/Tau5 ratios, rather increased the AT8/GAPDH, AT180/GAPDH, PHF-1/GAPDH and Tau5/GAPDH ratios. The rationale for using the LPS-induced systemic inflammation model is that it is a quick and convenient model to evaluate the effects of IL-1RAP deficiency on inflammation-induced tau pathology.

In the IL-1RAcP knockout validation, we observed three bands with the most bottom band being a doublet. The bad with the highest molecular weight close to 98 KD corresponds to the neuronal isoform. The middle band around 80 KD corresponds to the global isoform. And the bottom doublet band could be representative of the secreted isoform of the protein but requires further confirmation. IL-1RAcP has several putative glycosylation sites and undergoes glycosylation (Azimzadeh Irani & Ejtehadi, 2022), which increases its molecular weight. In addition, IL-1RAcP is also a substrate for kinases, ubiquitinase, and possibly other post-translational modifications. For example, E3 ubiquitin ligase membrane-associated RING-CH (MARCH8) has been shown to interact with IL-1RAcP and targets Lys512 for K48-linked polyubiquination and degradation, thereby MARCH8 serves as a negative regulator of IL-1β signaling (Chen et al., 2012). Post-translational modifications of IL-1RAcP are an emerging area of research, and only limited literature is available, which would provide scientific rationale for different bands of IL-1RAcP that we report here.

LPS-injected IL-1RAcP deficient mice showed a significantly lower AT8/Tau5 ratio but no change in AT180/Tau5 or PHF-1/Tau5, and a significant reduction in AT8/GAPDH and AT180/GAPDH, but not in PHF-1/GAPDH when compared to LPS-injected wild type mice after 24 hours. These results were consistent with our prior study (Bhaskar et al., 2010), where IL-1R1 deficient mice showed significantly reduced AT8 levels in an LPS-induced model of systemic inflammation, albeit the LPS used was 10 mg/kg b.w., versus 3.5 mg/kg b.w., much lower in the present study. These results suggest that targeting either IL-1R1 or IL-1RAcP is protective against LPS-induced tau hyperphosphorylation. The AT8 sites are commonly phosphorylated early in tau pathology (Barthélemy et al., 2020; Morris et al., 2020) and are highly responsive to inflammatory stimuli, such as LPS (Bhaskar et al., 2010; Kitazawa et al., 2005; Lee et al., 2010; Maphis et al., 2015; Su et al., 1994; Sy et al., 2011; Yang et al., 2014). IL-1RAcP knockout may impact signaling pathways (e.g., p38 MAPK) that specifically regulate tau phosphorylation on these residues. While AT180 (phospho-Thr231) is also linked to early tau pathology (Barthélemy et al., 2020), it may be mediated by distinct kinases (e.g., CDK5), which could be differentially affected by IL-1RAcP deficiency in a model of LPS-induced tauopathy. The PHF-1 (phospho-Ser396/Ser404) is a later-stage tauopathy marker, and a 24-hour LPS treatment may not strongly induce phosphorylation at these sites (Augustinack et al., 2002; Spires-Jones et al., 2009; Wesseling et al., 2020). Alternatively, IL-1RAcP knockout may selectively modulate kinases that phosphorylate early residues (e.g., AT8) but not those targeting PHF-1 (Cho & Johnson, 2003, 2004; Wesseling et al., 2020). Furthermore, protein phosphatases (e.g., PP1, PP2A) may be differentially activated in IL-1RAcP knockout mice, selectively dephosphorylating PHF-1 sites while leaving AT8 more affected (Stoothoff & Johnson, 2005), which may need to be explored in the future. Finally, in mouse tau, the PHF-1 is relatively more phosphorylated compared to those in human brains and when assessing this site phosphorylation, it is important to perform relative analyses compared to control groups.

**Figure 4.**
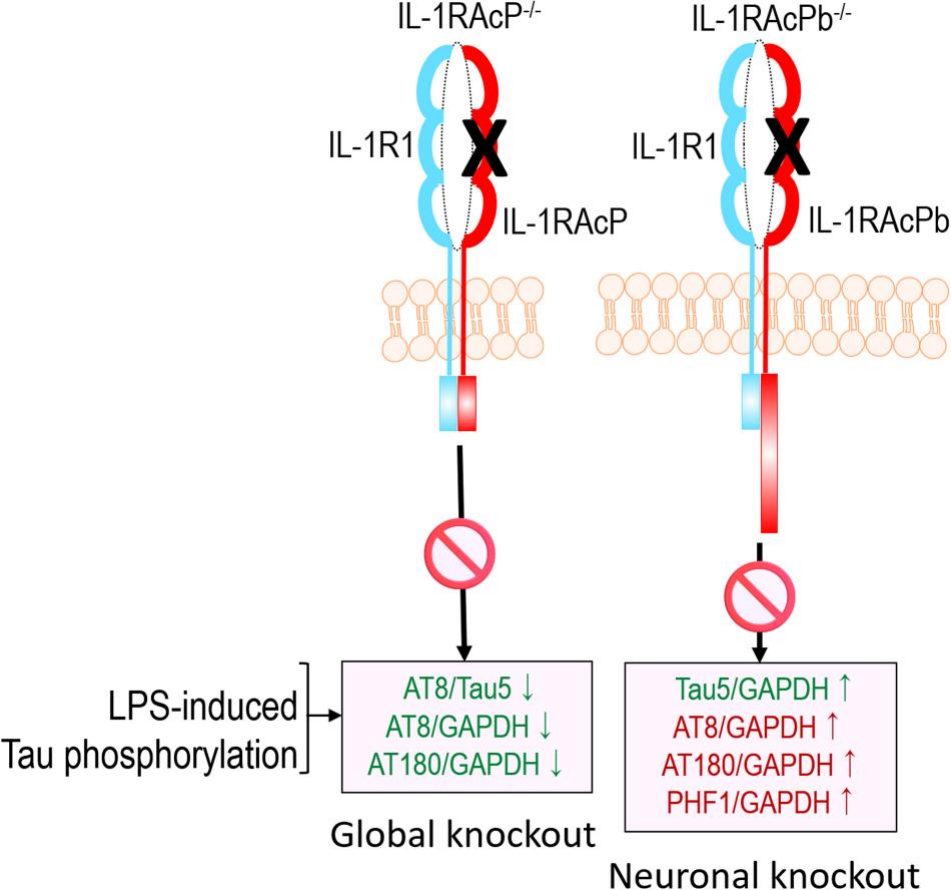
Working model showing the summary of IL-1RAcP and IL-1RAcPb deficiency outcome in the LPS-induced model of systemic inflammation. When mice injected with LPS, global IL-1RAcP knock out results in the reduction of AT8/Tau5, AT8/GAPDH, and AT180/GAPDH. LPS-injection to neuron-specific IL-1RAcPb^-/-^ mice show increased levels of Tau5/GAPDH and increased levels of AT8/GAPDH, AT180/GAPDH, and PHF-1/GAPDH.

When we challenged neuron-specific IL-1RAcPb deficient mice with LPS, unlike the global IL-1RAcP knockout mice, the *Il1rapb^-/-^* mice exhibited a modest reduction in AT8/Tau5, AT180/Tau5 and PHF-1/Tau5 ratios upon LPS challenge compared to LPS-injected WT mice (Figure 3B and C). However, AT8/GAPDH, AT180/GAPDH, PHF-1/GAPDH and Tau5/GAPDH levels were significantly elevated in LPS-injected IL-1RAcPb knockout mice compared to their WT counterparts. A higher Tau5/GAPDH ratio indicates an elevated total tau protein, which could be because of either increased tau expression or reduced tau degradation in response to IL-1RAcPb knockout in the model of systemic inflammation. Alternatively, elevated total tau also indicates that IL-1RAcPb deficiency prevents neurons from dying from LPS challenge and, therefore, shows elevated total tau. Whether or not this increase in total tau in LPS-injected *Il1rapb^-/-^* mice may reflect reduced neuronal loss, enhanced tau expression or increased stability of tau in the cells should be investigated in future studies (Figure 4). Nonetheless, the current study provides first direct evidence that removal of all isoforms of IL-1RAcP in IL-1RAcP global knockout mice is a better strategy to reduce inflammation-induced tau hyperphosphorylation rather than genetic removal of only neuron-specific IL-1RAcPb – despite tau being a neuronal protein. It also suggests the possibility that non-neuronal isoform of IL-1RAcP contributes to LPS-induced tau pathology. We have previously demonstrated that LPS, which causes neuroinflammation through toll-like receptor-4 (TLR-4), can trigger tau phosphorylation in neurons (Bhaskar et al., 2010). Conversely, in cases of tauopathies driven by the splice-site mutations in *MAPT,* which alters the ratio of 3-microtubule repeat tau (3 R-Tau) to 4 R-Tau and sufficient to drive frontotemporal dementias, elevated tau may mean that LPS-induced model may be increasing the production of more tau in IL-1RAcPb knockout mice. Prior studies have demonstrated that IL-1RAcP in general (and neuron-specific IL-1RAcPb isoform in particular) plays a role in maintaining neuronal homeostasis, long-term potentiation (LTP), and sleep cycle under physiological conditions (Davis et al., 2015; Fang et al., 1998; Taishi et al., 2012; Wang et al., 2007). However, it is unclear what role IL-1RAcPb plays during inflammatory conditions in neurons. Many of these possibilities need to be tested experimentally in future studies. Based on our study, we conclude that IL-1RAcPb deficiency might not significantly impact the cell in the context of tau pathology because the Tau5/GAPDH ratio is elevated while the AT8/Tau5, AT180/Tau5, and PHF-1/Tau5 show no significant changes. Together with the rs12053868 SNP reported in Ramanan et al., we could speculate that there might be a relationship between the total tau levels (neuroprotection/preventing neuronal atrophy) we observed in our study in LPS-injected IL-1RAcPb knockout mice and a likelihood of upregulation of IL-1RAcP/IL-1RAcPb activity in patients carrying the rs12053868 AD risk SNP. This possibility must be further validated in future studies.

In conclusion, we show that global and neuron-specific IL-1RAcP isoform deficiency regulates tau hyperphosphorylation in a mouse model of LPS-induced tauopathy. Given that the LPS model of systemic inflammation may involve cytokines and other immune pathways, it is important to validate the findings from this study in a mouse model of neurodegenerative tauopathies, such as PS19 mice and human subjects carrying rs12053868 AD risk alleles.

## Supplementary Material

Dadras_etal_SI_Revisedclean.docx

## References

[CIT0001] Alkam, T., Nitta, A., Mizoguchi, H., Saito, K., Seshima, M., Itoh, A., Yamada, K., & Nabeshima, T. (2008). Restraining tumor necrosis factor-alpha by thalidomide prevents the Amyloid beta-induced impairment of recognition memory in mice. *Behavioural Brain Research*, *189*(1), 100–106. 10.1016/j.bbr.2007.12.014 18325608

[CIT0002] Allan, D., Fairlie-Clarke, K. J., Elliott, C., Schuh, C., Barnett, S. C., Lassmann, H., Linnington, C., & Jiang, H.-R. (2016). Role of IL-33 and ST2 signalling pathway in multiple sclerosis: Expression by oligodendrocytes and inhibition of myelination in central nervous system. *Acta Neuropathologica Communications*, *4*(1), 75. 10.1186/s40478-016-0344-1 27455844 PMC4960877

[CIT0003] Augustinack, J. C., Schneider, A., Mandelkow, E. M., & Hyman, B. T. (2002). Specific tau phosphorylation sites correlate with severity of neuronal cytopathology in Alzheimer’s disease. *Acta Neuropathologica*, *103*(1), 26–35. 10.1007/s004010100423 11837744

[CIT0004] Azimzadeh Irani, M., & Ejtehadi, M. R. (2022). Glycan-mediated functional assembly of IL-1RI: structural insights into completion of the current description for immune response. *Journal of Biomolecular Structure & Dynamics*, *40*(6), 2575–2585. 10.1080/07391102.2020.1841027 33124956

[CIT0005] Barthélemy, N. R., Li, Y., Joseph-Mathurin, N., Gordon, B. A., Hassenstab, J., Benzinger, T. L. S., Buckles, V., Fagan, A. M., Perrin, R. J., Goate, A. M., Morris, J. C., Karch, C. M., Xiong, C., Allegri, R., Mendez, P. C., Berman, S. B., Ikeuchi, T., Mori, H., Shimada, H., … McDade, E. (2020). A soluble phosphorylated tau signature links tau, amyloid and the evolution of stages of dominantly inherited Alzheimer’s disease. *Nature Medicine*, *26*(3), 398–407. 10.1038/s41591-020-0781-z PMC7309367 32161412

[CIT0006] Batista, C. R. A., Gomes, G. F., Candelario-Jalil, E., Fiebich, B. L., & de Oliveira, A. C. P. (2019). Lipopolysaccharide-induced neuroinflammation as a bridge to understand neurodegeneration. *International Journal of Molecular Sciences*, *20*(9), 2293. 10.3390/ijms20092293 31075861 PMC6539529

[CIT0007] Bemiller, S. M., Maphis, N. M., Formica, S. V., Wilson, G. N., Miller, C. M., Xu, G., Kokiko-Cochran, O. N., Kim, K.-W., Jung, S., Cannon, J. L., Crish, S. D., Cardona, A. E., Lamb, B. T., & Bhaskar, K. (2018). Genetically enhancing the expression of chemokine domain of CX3CL1 fails to prevent tau pathology in mouse models of tauopathy. *Journal of Neuroinflammation*, *15*(1), 278. 10.1186/s12974-018-1310-6 30253780 PMC6154806

[CIT0008] Bhaskar, K., Konerth, M., Kokiko-Cochran, O. N., Cardona, A., Ransohoff, R. M., & Lamb, B. T. (2010). Regulation of tau pathology by the microglial fractalkine receptor. *Neuron*, *68*(1), 19–31. 10.1016/j.neuron.2010.08.023 20920788 PMC2950825

[CIT0009] Brough, D., & Denes, A. (2015). Interleukin-1α and brain inflammation. *IUBMB Life*, *67*(5), 323–330. 10.1002/iub.1377 25906979

[CIT0010] Byrne, J., Baker, K., Houston, A., & Brint, E. (2021). IL-36 cytokines in inflammatory and malignant diseases: Not the new kid on the block anymore. *Cellular and Molecular Life Sciences*, *78*(17-18), 6215–6227. 10.1007/s00018-021-03909-4 34365521 PMC8429149

[CIT0011] Chapuis, J., Hot, D., Hansmannel, F., Kerdraon, O., Ferreira, S., Hubans, C., Maurage, C. A., Huot, L., Bensemain, F., Laumet, G., Ayral, A. M., Fievet, N., Hauw, J. J., DeKosky, S. T., Lemoine, Y., Iwatsubo, T., Wavrant-Devrièze, F., Dartigues, J. F., Tzourio, C., … Lambert, J. C. (2009). Transcriptomic and genetic studies identify IL-33 as a candidate gene for Alzheimer’s disease. *Molecular Psychiatry*, *14*(11), 1004–1016. 10.1038/mp.2009.10 19204726 PMC2860783

[CIT0012] Chen, R., Li, M., Zhang, Y., Zhou, Q., & Shu, H. B. (2012). The E3 ubiquitin ligase MARCH8 negatively regulates IL-1β-induced NF-κB activation by targeting the IL1RAP coreceptor for ubiquitination and degradation. *Proceedings of the National Academy of Sciences of the United States of America*, *109*(35), 14128–14133. 10.1073/pnas.1205246109 22904187 PMC3435212

[CIT0013] Chen, W.-J., Yu, X., Yuan, X.-R., Chen, B.-J., Cai, N., Zeng, S., Sun, Y.-S., & Li, H.-W. (2021). The role of IL-36 in the pathophysiological processes of autoimmune diseases. *Frontiers in Pharmacology*, *12*, 727956. 10.3389/fphar.2021.727956 34675805 PMC8523922

[CIT0014] Cho, J. H., & Johnson, G. V. W. (2003). Glycogen synthase kinase 3beta phosphorylates tau at both primed and unprimed sites. Differential impact on microtubule binding. *The Journal of Biological Chemistry*, *278*(1), 187–193. 10.1074/jbc.M206236200 12409305

[CIT0015] Cho, J. H., & Johnson, G. V. W. (2004). Primed phosphorylation of tau at Thr231 by glycogen synthase kinase 3beta (GSK3beta) plays a critical role in regulating tau’s ability to bind and stabilize microtubules. *Journal of Neurochemistry*, *88*(2), 349–358. 10.1111/j.1471-4159.2004.02155.x 14690523

[CIT0016] Creekmore, B. C., Watanabe, R., & Lee, E. B. (2024). Neurodegenerative disease tauopathies. *Annual Review of Pathology*, *19*(1), 345–370. 10.1146/annurev-pathmechdis-051222-120750 PMC11009985 37832941

[CIT0017] Cruchaga, C., Kauwe, J. S. K., Harari, O., Jin, S. C., Cai, Y., Karch, C. M., Benitez, B. A., Jeng, A. T., Skorupa, T., Carrell, D., Bertelsen, S., Bailey, M., McKean, D., Shulman, J. M., De Jager, P. L., Chibnik, L., Bennett, D. A., Arnold, S. E., Harold, D., … Goate, A. M. (2013). GWAS of cerebrospinal fluid tau levels identifies risk variants for Alzheimer’s disease. *Neuron*, *78*(2), 256–268. 10.1016/j.neuron.2013.02.026 23562540 PMC3664945

[CIT0018] Cullinan, E. B., Kwee, L., Nunes, P., Shuster, D. J., Ju, G., McIntyre, K. W., Chizzonite, R. A., & Labow, M. A. (1998). IL-1 receptor accessory protein is an essential component of the IL-1 receptor. *The Journal of Immunology*, *161*(10), 5614–5620. 10.4049/jimmunol.161.10.5614 9820540

[CIT0019] Dani, M., Wood, M., Mizoguchi, R., Fan, Z., Walker, Z., Morgan, R., Hinz, R., Biju, M., Kuruvilla, T., Brooks, D. J., & Edison, P. (2018). Microglial activation correlates in vivo with both tau and amyloid in Alzheimer’s disease. *Brain*, *141*(9), 2740–2754. 10.1093/brain/awy188 30052812

[CIT0020] Davidson, R., Krider, R. I., Borsellino, P., Noorda, K., Alhwayek, G., & Vida, T. A. (2023). Untangling tau: Molecular insights into neuroinflammation, pathophysiology, and emerging immunotherapies. *Current Issues in Molecular Biology*, *45*(11), 8816–8839. 10.3390/cimb45110553 37998730 PMC10670294

[CIT0021] Davis, C. J., Dunbrasky, D., Oonk, M., Taishi, P., Opp, M. R., & Krueger, J. M. (2015). The neuron-specific interleukin-1 receptor accessory protein is required for homeostatic sleep and sleep responses to influenza viral challenge in mice. *Brain, Behavior, and Immunity*, *47*, 35–43. 10.1016/j.bbi.2014.10.013 25449578 PMC4418942

[CIT0022] Dursun, E., Gezen-Ak, D., Hanağası, H., Bilgiç, B., Lohmann, E., Ertan, S., Atasoy, İ. L., Alaylıoğlu, M., Araz, Ö. S., Önal, B., Gündüz, A., Apaydın, H., Kızıltan, G., Ulutin, T., Gürvit, H., & Yılmazer, S. (2015). The interleukin 1 alpha, interleukin 1 beta, interleukin 6 and alpha-2-macroglobulin serum levels in patients with early or late onset Alzheimer’s disease, mild cognitive impairment or Parkinson’s disease. *Journal of Neuroimmunology*, *283*, 50–57. 10.1016/j.jneuroim.2015.04.014 26004156

[CIT0023] Fang, J., Wang, Y., & Krueger, J. M. (1998). Effects of interleukin-1 beta on sleep are mediated by the type I receptor. *The American Journal of Physiology*, *274*(3), R655–R660. 10.1152/ajpregu.1998.274.3.R655 9530230

[CIT0024] Fields, J. K., Günther, S., & Sundberg, E. J. (2019). Structural basis of IL-1 family cytokine signaling. *Frontiers in Immunology*, *10*, 1412. 10.3389/fimmu.2019.01412 31281320 PMC6596353

[CIT0025] Ghosh, S., Wu, M. D., Shaftel, S. S., Kyrkanides, S., LaFerla, F. M., Olschowka, J. A., & O’Banion, M. K. (2013). Sustained interleukin-1β overexpression exacerbates tau pathology despite reduced amyloid burden in an Alzheimer’s mouse model. *The Journal of Neuroscience*, *33*(11), 5053–5064. 10.1523/JNEUROSCI.4361-12.2013 23486975 PMC3637949

[CIT0026] Italiani, P., Puxeddu, I., Napoletano, S., Scala, E., Melillo, D., Manocchio, S., Angiolillo, A., Migliorini, P., Boraschi, D., Vitale, E., & Di Costanzo, A. (2018). Circulating levels of IL-1 family cytokines and receptors in Alzheimer’s disease: New markers of disease progression? *Journal of Neuroinflammation*, *15*(1), 342. 10.1186/s12974-018-1376-1 30541566 PMC6292179

[CIT0027] Jensen, L. E., Muzio, M., Mantovani, A., & Whitehead, A. S. (2000). IL-1 signaling cascade in liver cells and the involvement of a soluble form of the IL-1 receptor accessory protein. *Journal of Immunology*, *164*(10), 5277–5286. 10.4049/jimmunol.164.10.5277 10799889

[CIT0028] Jiang, S., Maphis, N. M., Binder, J., Chisholm, D., Weston, L., Duran, W., Peterson, C., Zimmerman, A., Mandell, M. A., Jett, S. D., Bigio, E., Geula, C., Mellios, N., Weick, J. P., Rosenberg, G. A., Latz, E., Heneka, M. T., & Bhaskar, K. (2021). Proteopathic tau primes and activates interleukin-1β via myeloid-cell-specific MyD88- and NLRP3-ASC-inflammasome pathway. *Cell Reports*, *36*(12), 109720. 10.1016/j.celrep.2021.109720 34551296 PMC8491766

[CIT0029] Jones, L., Holmans, P. A., Hamshere, M. L., Harold, D., Moskvina, V., Ivanov, D., Pocklington, A., Abraham, R., Hollingworth, P., Sims, R., Gerrish, A., Pahwa, J. S., Jones, N., Stretton, A., Morgan, A. R., Lovestone, S., Powell, J., Proitsi, P., Lupton, M. K., … Williams, J. (2010). Genetic evidence implicates the immune system and cholesterol metabolism in the aetiology of Alzheimer’s disease. *PLoS One*, *5*(11), e13950. 10.1371/journal.pone.0013950 21085570 PMC2981526

[CIT0030] Kitazawa, M., Cheng, D., Tsukamoto, M. R., Koike, M. A., Wes, P. D., Vasilevko, V., Cribbs, D. H., & LaFerla, F. M. (2011). Blocking IL-1 signaling rescues cognition, attenuates tau pathology, and restores neuronal β-catenin pathway function in an Alzheimer’s disease model. *Journal of Immunology*, *187*(12), 6539–6549. 10.4049/jimmunol.1100620 PMC4072218 22095718

[CIT0031] Kitazawa, M., Oddo, S., Yamasaki, T. R., Green, K. N., & LaFerla, F. M. (2005). Lipopolysaccharide-induced inflammation exacerbates tau pathology by a cyclin-dependent kinase 5-mediated pathway in a transgenic model of Alzheimer’s disease. *The Journal of Neuroscience*, *25*(39), 8843–8853. 10.1523/JNEUROSCI.2868-05.2005 16192374 PMC6725603

[CIT0032] Langworth-Green, C., Patel, S., Jaunmuktane, Z., Jabbari, E., Morris, H., Thom, M., Lees, A., Hardy, J., Zandi, M., & Duff, K. (2023). Chronic effects of inflammation on tauopathies. *The Lancet. Neurology*, *22*(5), 430–442. 10.1016/S1474-4422(23)00038-8 37059510

[CIT0033] Lee, D. C., Rizer, J., Selenica, M.-L. B., Reid, P., Kraft, C., Johnson, A., Blair, L., Gordon, M. N., Dickey, C. A., & Morgan, D. (2010). LPS-induced inflammation exacerbates phospho-tau pathology in rTg4510 mice. *Journal of Neuroinflammation*, *7*(1), 56. 10.1186/1742-2094-7-56 20846376 PMC2949628

[CIT0034] Lu, H. L., Yang, C. Y., Chen, H. C., Hung, C. S., Chiang, Y. C., & Ting, L. P. (2008). A novel alternatively spliced interleukin-1 receptor accessory protein mIL-1RAcP687. *Molecular Immunology*, *45*(5), 1374–1384. 10.1016/j.molimm.2007.09.002 17949817

[CIT0035] Luheshi, N. M., Kovács, K. J., Lopez-Castejon, G., Brough, D., & Denes, A. (2011). Interleukin-1α expression precedes IL-1β after ischemic brain injury and is localised to areas of focal neuronal loss and penumbral tissues. *Journal of Neuroinflammation*, *8*(1), 186. 10.1186/1742-2094-8-186 22206506 PMC3259068

[CIT0036] Maphis, N. M., Hulse, J., Peabody, J., Dadras, S., Whelpley, M. J., Kandath, M., Wilson, C., Hobson, S., Thompson, J., Poolsup, S., Beckman, D., Ott, S. P., Watanabe, J. W., Usachenko, J. L., Van Rompay, K. K., Morrison, J., Selwyn, R., Rosenberg, G., Knoefel, J., Chackerian, B., & Bhaskar, K. (2025). Targeting of phosphorylated tau at threonine 181 by a Qβ virus‐like particle vaccine is safe, highly immunogenic, and reduces disease severity in mice and rhesus macaques. *Alzheimer’s & Dementia*, *21*(3), e70101. 10.1002/alz.70101 PMC11947757 40145301

[CIT0037] Maphis, N., Xu, G., Kokiko-Cochran, O. N., Cardona, A. E., Ransohoff, R. M., Lamb, B. T., & Bhaskar, K. (2015). Loss of tau rescues inflammation-mediated neurodegeneration. *Frontiers in Neuroscience*, *9*, 196. 10.3389/fnins.2015.00196 26089772 PMC4452825

[CIT0038] Maphis, N., Xu, G., Kokiko-Cochran, O. N., Jiang, S., Cardona, A., Ransohoff, R. M., Lamb, B. T., & Bhaskar, K. (2015). Reactive microglia drive tau pathology and contribute to the spreading of pathological tau in the brain. *Brain*, *138*(Pt 6), 1738–1755. 10.1093/brain/awv081 25833819 PMC4542622

[CIT0039] Masoumi, J., Vakilian, A., Sayadi, A., Shekari, N., & Khorramdelazad, H. (2020). Assessing the gene expression of interleukin-36 in Alzheimer’s patients. *Gene Reports*, *21*, 100823. 10.1016/j.genrep.2020.100823

[CIT0040] Mendiola, A. S., & Cardona, A. E. (2018). The IL-1β phenomena in neuroinflammatory diseases. *Journal of Neural Transmission*, *125*(5), 781–795. 10.1007/s00702-017-1732-9 28534174 PMC5699978

[CIT0041] Metcalfe, M. J., & Figueiredo-Pereira, M. E. (2010). Relationship between tau pathology and neuroinflammation in Alzheimer’s disease. *The Mount Sinai Journal of Medicine*, *77*(1), 50–58. 10.1002/msj.20163 20101714 PMC2904237

[CIT0042] Morris, S. L., Tsai, M.-Y., Aloe, S., Bechberger, K., König, S., Morfini, G., & Brady, S. T. (2020). Defined tau phosphospecies differentially inhibit fast axonal transport through activation of two independent signaling pathways. *Frontiers in Molecular Neuroscience*, *13*, 610037. 10.3389/fnmol.2020.610037 33568975 PMC7868336

[CIT0043] Rachal Pugh, C., Fleshner, M., Watkins, L. R., Maier, S. F., & Rudy, J. W. (2001). The immune system and memory consolidation: A role for the cytokine IL-1β. *Neuroscience and Biobehavioral Reviews*, *25*(1), 29–41. 10.1016/S0149-7634(00)00048-8 11166076

[CIT0044] Ramanan, V. K., Risacher, S. L., Nho, K., Kim, S., Shen, L., McDonald, B. C., Yoder, K. K., Hutchins, G. D., West, J. D., Tallman, E. F., Gao, S., Foroud, T. M., Farlow, M. R., De Jager, P. L., Bennett, D. A., Aisen, P. S., Petersen, R. C., Jack, C. R., Toga, A. W., … Saykin, A. J. (2015). GWAS of longitudinal amyloid accumulation on 18F-florbetapir PET in Alzheimer’s disease implicates microglial activation gene IL1RAP. *Brain*, *138*(Pt 10), 3076–3088. 10.1093/brain/awv231 26268530 PMC4671479

[CIT0045] Reverchon, F., de Concini, V., Larrigaldie, V., Benmerzoug, S., Briault, S., Togbé, D., Ryffel, B., Quesniaux, V. F. J., & Menuet, A. (2020). Hippocampal interleukin-33 mediates neuroinflammation-induced cognitive impairments. *Journal of Neuroinflammation*, *17*(1), 268. 10.1186/s12974-020-01939-6 32917228 PMC7488545

[CIT0046] Rossi, S., Motta, C., Studer, V., Macchiarulo, G., Volpe, E., Barbieri, F., Ruocco, G., Buttari, F., Finardi, A., Mancino, R., Weiss, S., Battistini, L., Martino, G., Furlan, R., Drulovic, J., & Centonze, D. (2014). Interleukin-1β causes excitotoxic neurodegeneration and multiple sclerosis disease progression by activating the apoptotic protein p53. *Molecular Neurodegeneration*, *9*(1), 56. 10.1186/1750-1326-9-56 25495224 PMC4292815

[CIT0047] Sims, J. E., & Smith, D. E. (2010). The IL-1 family: Regulators of immunity. *Nature Reviews. Immunology*, *10*(2), 89–102. 10.1038/nri2691 20081871

[CIT0048] Smith, D. E., Lipsky, B. P., Russell, C., Ketchem, R. R., Kirchner, J., Hensley, K., Huang, Y., Friedman, W. J., Boissonneault, V., Plante, M.-M., Rivest, S., & Sims, J. E. (2009). A central nervous system-restricted isoform of the interleukin-1 receptor accessory protein modulates neuronal responses to interleukin-1. *Immunity*, *30*(6), 817–831. 10.1016/j.immuni.2009.03.020 19481478 PMC4103746

[CIT0049] Spires-Jones, T. L., Stoothoff, W. H., de Calignon, A., Jones, P. B., & Hyman, B. T. (2009). Tau pathophysiology in neurodegeneration: a tangled issue. *Trends in Neurosciences*, *32*(3), 150–159. 10.1016/j.tins.2008.11.007 19162340

[CIT0050] Stoothoff, W. H., & Johnson, G. V. W. (2005). Tau phosphorylation: Physiological and pathological consequences. *Biochimica et Biophysica Acta*, *1739*(2-3), 280–297. 10.1016/j.bbadis.2004.06.017 15615646

[CIT0051] Su, J. H., Cummings, B. J., & Cotman, C. W. (1994). Early phosphorylation of tau in Alzheimer’s disease occurs at Ser-202 and is preferentially located within neurites. *Neuroreport*, *5*(17), 2358–2362. 10.1097/00001756-199411000-00037 7533559

[CIT0052] Sy, M., Kitazawa, M., Medeiros, R., Whitman, L., Cheng, D., Lane, T. E., & Laferla, F. M. (2011). Inflammation induced by infection potentiates tau pathological features in transgenic mice. *The American Journal of Pathology*, *178*(6), 2811–2822. 10.1016/j.ajpath.2011.02.012 21531375 PMC3124234

[CIT0053] Taishi, P., Davis, C. J., Bayomy, O., Zielinski, M. R., Liao, F., Clinton, J. M., Smith, D. E., & Krueger, J. M. (2012). Brain-specific interleukin-1 receptor accessory protein in sleep regulation. *Journal of Applied Physiology*, *112*(6), 1015–1022. 10.1152/japplphysiol.01307.2011 22174404 PMC3311656

[CIT0054] Wang, J. Z., Grundke-Iqbal, I., & Iqbal, K. (2007). Kinases and phosphatases and tau sites involved in Alzheimer neurofibrillary degeneration. *The European Journal of Neuroscience*, *25*(1), 59–68. 10.1111/j.1460-9568.2006.05226.x 17241267 PMC3191918

[CIT0055] Wesseling, H., Mair, W., Kumar, M., Schlaffner, C. N., Tang, S., Beerepoot, P., Fatou, B., Guise, A. J., Cheng, L., Takeda, S., Muntel, J., Rotunno, M. S., Dujardin, S., Davies, P., Kosik, K. S., Miller, B. L., Berretta, S., Hedreen, J. C., Grinberg, L. T., … Steen, J. A. (2020). Tau PTM profiles identify patient heterogeneity and stages of Alzheimer’s disease. *Cell*, *183*(6), 1699–1713.e13. 10.1016/j.cell.2020.10.029 33188775 PMC8168922

[CIT0056] Weston, L. L., Jiang, S., Chisholm, D., Jantzie, L. L., & Bhaskar, K. (2021). Interleukin-10 deficiency exacerbates inflammation-induced tau pathology. *Journal of Neuroinflammation*, *18*(1), 161. 10.1186/s12974-021-02211-1 34275478 PMC8286621

[CIT0057] When are mice considered old?. (2023, July 24). The Jackson Laboratory. Retrieved July 23, 2023, from https://www.jax.org/news-and-insights/jax-blog/2017/November/when-are-mice-considered-old

[CIT0058] Xiong, Z., Thangavel, R., Kempuraj, D., Yang, E., Zaheer, S., & Zaheer, A. (2014). Alzheimer’s disease: Evidence for the expression of interleukin-33 and its receptor ST2 in the brain. *Journal of Alzheimer’s Disease*, *40*(2), 297–308. 10.3233/JAD-132081 PMC4015800 24413615

[CIT0059] Yang, S., Gong, Q., Wu, Q., Li, F., Lu, Y., & Shi, J. (2014). Alkaloids enriched extract from *Dendrobium nobile* Lindl. attenuates tau protein hyperphosphorylation and apoptosis induced by lipopolysaccharide in rat brain. *Phytomedicine*, *21*(5), 712–716. 10.1016/j.phymed.2013.10.026 24268296

[CIT0060] Yoshiyama, Y., Higuchi, M., Zhang, B., Huang, S.-M., Iwata, N., Saido, T. C., Maeda, J., Suhara, T., Trojanowski, J. Q., & Lee, V. M.-Y. (2007). Synapse loss and microglial activation precede tangles in a P301S tauopathy mouse model. *Neuron*, *53*(3), 337–351. 10.1016/j.neuron.2007.01.010 17270732

[CIT0061] Yuan, Z. C., Xu, W. D., Liu, X. Y., Liu, X. Y., Huang, A. F., & Su, L. C. (2019). Biology of IL-36 signaling and its role in systemic inflammatory diseases. *Frontiers in Immunology*, *10*, 2532. 10.3389/fimmu.2019.02532 31736959 PMC6839525

[CIT0062] Zhang, Z.-Y., Li, J., Ye, Q., Dong, Y., Bao, G.-M., Shen, Y.-K., Weng, J.-F., Luo, L.-F., & Cen, M. (2019). Usefulness of serum interleukin-33 as a prognostic marker of severe traumatic brain injury. *Clinica Chimica Acta*, *497*, 6–12. 10.1016/j.cca.2019.07.008 31279693

