# Supplementary material for: Global Deficiency of Alzheimer’s Disease Risk Gene Il1rap Reduces Pathological Tau in a Mouse Model of Systemic Inflammation
Source: ASN Neuro. 2025 Dec 6;17(1):2598310. doi: 10.1080/17590914.2025.2598310 (PMC12688222; doi:10.1080/17590914.2025.2598310)
Supplement: Dadras_etal_SI_Revisedclean.docx [file TASN_A_2598310_SM7246.docx]

**Supplemental Table S1**

| **REAGENT NAME** | **SOURCE** | **IDENTIFIER** |
| --- | --- | --- |
| **Antibodies** | | |
| Rabbit monoclonal- IL-1RAP | Invitrogen | Cat# MA5-4146  RRID: AB_3094302 |
| Mouse monoclonal phospho-T231 tau (AT180) | ThermoFisher | Cat # MN1040;  RRID: AB_223649 |
| Mouse monoclonal phospho-S202 tau (AT8) | ThermoFisher | Cat # MN1020;  RRID: AB_223647 |
| Mouse monoclonal anti-GAPDH | Millipore | Cat # CB1001-500UG;  RRID: AB_2107426 |
| Mouse monoclonal anti-phospho-S396/S404 tau (PHF1) | Gift from Dr. Peter Davies | ^1^ |
| Mouse monoclonal anti-Tau5 | ThermoFisher | Cat # AHB0042;  RRID: AB_1502093 |
| Peroxidase AffiniPure® Goat Anti-Mouse IgG | Jackson ImmunoResearch Laboratories, Inc. | Cat #115-035-146  RRID: AB_2307392 |
| Peroxidase AffiniPure® Goat Anti-Rabbit IgG | Jackson ImmunoResearch Laboratories, Inc. | Cat #111- 035-144  RRID: AB_2307391 |
| **Chemicals, Peptides, and Recombinant Proteins** |  |  |
| LPS | Sigma-Aldrich | Cat# L2880-25MG |
| 2-mercaptoethanol | Sigma-Aldrich |  |
| TRIzol™ Reagent | ThermoFisher | Cat # 15596026 |
| **Critical Commercial Assays** |  |  |
| High-Capacity cDNA Reverse Transcription Kit | ThermoFisher | Cat # 4368813 |
| One*Taq^®^* 2X Master Mix with Standard Buffer | New England Biolabs | Cat # M0482S |
| **Experimental Models: Organisms/Strains** |  |  |
| C57BL/6J mice | Jackson Laboratory | Cat # 000664;  RRID: IMSR_JAX:000664 |
| *Il1rap^-/-^* mice | Dr. James Krueger | ^2^ |
| *Il1rapb^-/-^* mice | Dr. James Krueger | ^3^ |
| **Software and Algorithms** |  |  |
| ImageJ | NIH | <https://imagej.net/>; RRID:SCR_003070 |
| AlphaEaseFC^TM^ | AlphaInnotech | <http://genetictechnologiesinc.com/alpha/alpha_ease_fc.htm> |
| Adobe Photoshop CC | Adobe | https://www.adobe.com/products/photoshop. html;  RRID: SCR_014199 |
| Prism | GraphPad | https://www.graphpad.com/scientific- software/prism/;  RRID: SCR_002798 |
| **Other** |  |  |
| Protease inhibitor cocktail | Sigma-Aldrich | Cat # P8340 |
| Phosphatase inhibitor cocktail | Sigma-Aldrich | Cat # P5726 |
| Tissue protein extraction reagent (TPER) | ThermoFisher | Cat # 78510 |
| Lithium dodecyl sulfate (LDS) | ThermoFisher | Cat # B0007 |
| Reducing Agent (RA) | ThermoFisher | Cat # NP0009 |
| NuPAGE™ 4-12% Bis-Tris Protein Gels  NuPAGE^TM^ 8% Bis-Tris Protein Gels | ThermoFisher/  Invitrogen | Cat # NP0335BOX  Cat # WB1002BX10 |
| PVDF Transfer Membranes, 0.2μm | ThermoFisher | Cat # 88520 |
| ECL substrate | ThermoFisher | Cat # 34577 |
| Prestained protein ladder | ThermoFisher | Cat # 26616 |
| Bovine serum albumin | Sigma | Cat # 9647 |
| Blotting grade blocker nonfat dry milk | Bio-Rad | Cat # 1706404XTU |
| ABC reagent | Vector Laboratories | Cat# PK-4000 |
| SIGMAFAST™ 3,3′-Diaminobenzidine tablets | Sigma-Aldrich | Cat# D4293 |
| Permount | Fischer Scientific | Cat# SP15-100 |
| PCR primer pairs |  |  |
| AcPb F: 5’-TGTTTCCTATGCAAGAAATGTGGAAGAAGAGG-3’ | | |
| AcPb R: 5’- ATGGGGTTGCTCAAGCGGACGGTACTCCAC -3’ | | |
| AcP KO region F: 5’-CATGTTGAGGAACACAACTTACTG -3’ | | |
| AcP KO region R: 5’-ACAACCCTTATACCAAGTGACC-3’ | | |
| α-actin F: 5’-CCGGGAGAAGATGACTCAAA-3’ | | |
| α-actin R: 5’-GTGGTCACGAAGGAATAGCC-3’ | | |

References

1. Greenberg SG, Davies P, Schein JD, Binder LI. Hydrofluoric acid-treated tau PHF proteins display the same biochemical properties as normal tau. *J Biol Chem*. 1992;267(1):564-569.

2. Smith DE, Lipsky BP, Russell C, et al. A central nervous system-restricted isoform of the interleukin-1 receptor accessory protein modulates neuronal responses to interleukin-1. *Immunity*. 2009;30(6):817-831. doi:10.1016/j.immuni.2009.03.020

3. Cullinan EB, Kwee L, Nunes P, et al. IL-1 Receptor Accessory Protein Is an Essential Component of the IL-1 Receptor. *The Journal of Immunology*. 1998;161(10):5614-5620. doi:10.4049/jimmunol.161.10.5614
